# The Importance of Using Multiple Approaches for Identifying Emerging Invasive Species: The Case of the Rasberry Crazy Ant in the United States

**DOI:** 10.1371/journal.pone.0045314

**Published:** 2012-09-19

**Authors:** Dietrich Gotzek, Seán G. Brady, Robert J. Kallal, John S. LaPolla

**Affiliations:** 1 Department of Entomology, National Museum of Natural History, Smithsonian Institution, Washington, District of Columbia, United States of America; 2 Department of Biological Sciences, Towson University, Towson, Maryland, United States of America; Field Museum of Natural History, United States

## Abstract

In the past decade, Houston, Texas has been virtually overrun by an unidentified ant species, the sudden appearance and enormous population sizes and densities of which have received national media attention. The Rasberry Crazy Ant, as it has become known due to its uncertain species status, has since spread to neighboring states and is still a major concern to pest control officials. Previous attempts at identifying this species have resulted in widely different conclusions in regards to its native range, source, and biology. We identify this highly invasive pest species as *Nylanderia fulva* (Mayr) using morphometric data measured from 14 characters, molecular sequence data consisting of 4,669 aligned nucleotide sites from six independent loci and comparison with type specimens. This identification will allow for the study and control of this emerging pest species to proceed unencumbered by taxonomic uncertainty. We also show that *N. fulva* has a much wider distribution than previously thought and has most likely invaded all of the Gulf Coast states.

## Introduction

Invasive pest species are a major cause and consequence of human-mediated global change and hence one of the major challenges facing human agriculture and biodiversity conservation [1], [2], [3]. The number of invasive species is expected to rise with growing global trade due to increased propagule pressure [4], [5]. The nature of founding events of invasions can be a vital determinant of their long-term success and impact, which is why they are generally intensely studied, often leading to important management decisions [5], [6], [7], [8].

While early detection and monitoring are important to the study, assessment and management of new invasions, a critical first step is the accurate identification of the invading taxon [9]. Correctly identified pest species allow for immediate control and management efforts without wasting valuable resources, placing the discipline of taxonomy at the forefront of invasive species research. Natural history collections thus represent an important resource for bioinvasion research as they not only contain historical specimen records documenting the spread of pest species [10], but are also invaluable references for proper species identification and study of intraspecific variation [11]. However, accurate identification of a new pest is not always easy. Many introduced insects belong to species complexes that contain many morphologically similar cryptic species. Subsequently, proper identification can be limited by a lack of taxonomic resources (modern keys), distinctive morphological characteristics, or even a muddled taxonomic history. Often morphometric [12] or molecular [13], [14], [15] data are used to identify the new invasive, although such singular approaches are potentially inadequate [16], [17]. To overcome these taxonomic obstacles, multiple approaches are ideal including the combination of morphometric and genetic data [17], [18], [19], and the utilization of type specimen material whenever possible.

Sudden, explosive outbreaks of unidentified pests are relatively rare, which is why the appearance of an unknown species of crazy ant in the genus *Nylanderia* (Hymenoptera: Formicidae) (formerly *Paratrechina* [20]) in 2002 near Houston, Texas, U.S.A. received extensive media coverage. Particularly worrisome was the preliminary observation that this crazy ant can successfully compete with and even displace the Red Imported Fire Ant, *Solenopsis invicta* Buren [21], one of the most costly invasive arthropods in the United States [22] and generally considered to be one of the worst invasive insects in the world [23]. The Texas and Federal Departments of Agriculture consider the pest to be a major concern for agriculture and have assigned a Best Management Practices Task Force to help control this pest. However, reliable estimates for the damage and potential impact of this new pest are still lacking, in part, because its taxonomic uncertainty has prevented any previous research or historical accounts from being used effectively.

**Figure 1:**
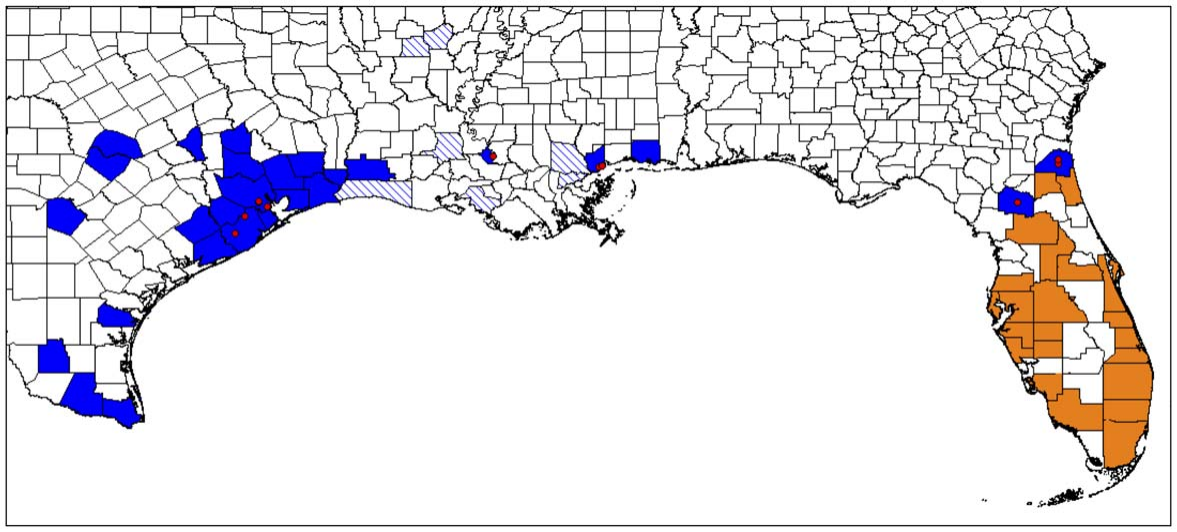
Reported distribution of the Rasberry Crazy Ant in the United States (in blue). The distribution of *Nylanderia cf. pubens* in Florida is given in orange, but we suspect that these may prove to be *N. fulva*. Counties highlighted with solid colors indicate verified occurrences, whereas hatched counties are unconfirmed reports. Red dots indicate collection sites for samples used in this study. The actual distribution of *N. fulva* in the United States is most likely to be more widespread.

Since its detection in Harris County, Texas, the new invasive has rapidly expanded its range and is now found in 21 counties in southeast Texas (http://urbanentomology.tamu.edu/ants/exotic_tx.cfm) and has recently been discovered from southwestern Mississippi [24] and Louisiana [25] (Fig. 1). Despite widespread attention, this invasive species has not been clearly identified [24]. Lacking a proper species name, it became known as the Rasberry Crazy Ant (RCA) for its discoverer, exterminator Tom Rasberry. Attempts at identification using barcoding [21] and morphometric analyses [21], [26] placed it near *N. pubens* (Forel), but could not exclude closely related species. The genus *Nylanderia* has a long history of taxonomic uncertainty in North America largely due to a lack of distinctive morphological characters in the worker caste [27]. A recent phylogeny placed it within a clade of mostly undescribed species of South American and Caribbean crazy ants [20], but this study was not designed to include sufficient sampling of RCA and related individuals in order to address its species status. The species designation of the RCA is still unresolved [21], [24], [26], [28], which is very problematic for biologically informed pest control and management.

We compared morphometric and molecular DNA sequence data of the RCA to 19 candidate conspecific *Nylanderia* species from North America, the Caribbean, and one introduced species from the Old World (*N. bourbonica*) to determine the species identity of the RCA. In particular, we focus on *N. pubens* and *N. fulva*, because they are both already established in the southeastern United States and are generally considered to be the best candidates for species identity of the RCA [21], [24], [26], [28]. We also describe a reliable and easily visible distinguishing morphological character to aid in identification of *N. fulva* and *N. pubens* based on comparison with the type material.

## Materials and Methods

### Specimen Information

Specimens collection information is given in Table 1 and Tables S1 and S2. All specimens were identified by JSL and RJK based on type material if the available material allowed unequivocal identification (see below). In particular, syntype material was examined for both *N. fulva* (12 workers; NHMW) and *N. pubens* (5 workers, 2 males, 1 queen; MHNG). Generally, we attempted to generate molecular and morphometric data for all specimens and to differentiate between *N. pubens* and *N. fulva* using males for all collection series. However, given the constraints of age, condition, and sex of material available to us, our ability to generate such data for every sample was limited. We initially reserved calling samples either *N. pubens* or *N. fulva* only if they were type specimens or if we were able to unambiguously assign them to either species based on male morphology (see below). DNA sequence data later allowed us to unequivocally assign most other indeterminate samples to *N. pubens* and *N. fulva*, leaving only seven Caribbean and three North American samples of unresolved taxonomic status (Table 1). Samples of *Nylanderia* that we could not unequivocally identify (as either *N. pubens* or *N. fulva*) were considered as *N. cf. pubens* if they were collected from the Caribbean or Florida (where *N. pubens* is well known to occur) or as RCA if collected from the invasive range (i.e., Texas and Mississippi). Specimens were borrowed from the following depositories:

**Table 1:** Species identification and collection localities of samples of *N. pubens*, *N. fulva*, and the RCA/*N. cf. pubens* used in this study.

Specimen	Collection locality	males	molecular	morphometrics
*N. fulva* syntypes	Brazil: Rio de Janeiro			X
*N. fulva* (RCA00)	USA: Texas: Harris Co.		X	
*N. fulva* (RCA01)	USA: LA: West Baton Rouge Co.	X	X	X
*N. fulva* (*cf. pubens*02)	USA: FL: Alachua Co.	X	X	
*N. fulva* (RCA05)	USA: MS: Hancock Co.	X		X
*N. fulva* (RCA07)	USA: TX: Harris Co.	X	X	X
*N. fulva* (RCA08)	USA: TX: Brazoria Co.	X		X
*N. fulva* (RCA09)	USA: TX: Harris Co.	X	X	X
*N. fulva* (*cf. pubens*13)	USVI: St. Croix: Southcentral		X	X
*N. fulva* (RCA21)	USA: TX: Harris Co.	X	X	X
*N. fulva* (*cf. pubens*22)	USA: FL: Duval Co.	X	X	X
*N. fulva* (*cf. pubens*23)	USA: FL: Alachua Co.		X	X
*N. fulva* (*fulva*24)	Paraguay: Neembucu	X	X	X
*N. fulva* (*fulva*25)	Paraguay: Canindeyu	X	X	
RCA03	USA: TX: Brazoria Co.			X
RCA06	USA: MS: Hancock Co.			X
*N. cf.* pubens04	USA: FL: Duval Co.			X
*N. cf. pubens*10	St. Kitts: St. Paul Capisterre Par.			X
*N. cf. pubens*11	USVI: St Croix: Southcentral			X
*N. cf. pubens*12	Barbados: St. Peter Par.			X
*N. cf. pubens*14	USVI: St. Croix: Northcentral			X
*N. cf. pubens*16	Anguilla: Junk’s Hole Bay			X
*N. cf. pubens*17	Barbados: St. Peter Par.			X
*N. cf. pubens*18	St. Lucia: Vieux Fort Quar.			X
*N. pubens* syntypes	St. Vincent: St. George Par.	X		X
*N. pubens* (*cf. pubens*15)	Anguilla: True Eyes Rd	X	X	X
*N. pubens* (*cf. pubens*19)	Anguilla: Lake’s Quarry		X	X
*N. pubens* (*cf. pubens*20)	St. Kitts: St. Mary Cayon Par.		X	X

All samples (except the types) were provisionally classified as the RCA if collected in the known range of the RCA or as *N. cf. pubens* if collected in Florida or the Caribbean (indicated in parentheses). Unequivocal species identifications were then based on males and/or molecular data. Specimens for which we were only able to generate morphometric data and no males were present remained indeterminate. Morphometric data in square brackets were excluded from DAPC due to missing data points.

ARCH: Archbold Biological Station, Florida, U.S.A.

JCTC: James C. Trager, private collection, U.S.A.

JKWC: Jim K. Wetterer, private collection, U.S.A.

LACM: Natural History Museum of Los Angeles County, U.S.A.

LRDC: Lloyd R. Davis, Jr., private collection, U.S.A.

NHMW: Naturhistorisches Museum, Wien, Austria.

MEMU: Mississippi Entomological Museum, Mississippi State, Mississippi, U.S.A.

MHNG: Muséum d’Histoire Naturelle, Geneva, Switzerland.

MCZC: Museum of Comparative Zoology, Harvard University, Cambridge, Massachusetts, U.S.A.

RSC: Rebecca Strecker, private collection, U.S.A.

TUBC: Towson University Biodiversity Center, Maryland, U.S.A.

UCDC: University of California, Davis, R.M. Bohart Museum of Entomology, California, U.S.A.

USNM: National Museum of Natural History, Washington, D.C., U.S.A.

WMC: William Mackay, private collection, U.S.A.

Vouchers of all specimens are deposited at the USNM. We report a new collection record for the RCA from Alachua and Duval Counties, Florida. Occurrence records for the RCA and *N. cf. pubens* in the United States were taken from Hooper-Bui [25], MacGown and Layton [24], the Center for Urban and Structural Entomology [29], and D. Oi (pers. comm. to DG).

### Morphometric Analysis

Nest averages were calculated for 14 standard morphometric measurements used in ant taxonomy (following LaPolla et al. [30], including profemur length and profemur width) from 1–4 workers (Table S1).

EL (Eye Length): maximum length of compound eye in full-face view.

GL (Gaster Length): the length of the gaster in lateral view from the anteriormost point of the first gastral segment (third abdominal segment) to the posteriormost point.

HL (Head Length): the length of the head proper, excluding the mandibles; measured in full-face view from the midpoint of the anterior clypeal margin to a line drawn across the posterior margin from its highest points (to accommodate species where the posterior margin is concave).

HW (Head Width): the maximum width of the head in full-face view (in males, portion of the eyes that extends past the lateral margins of the head is included).

MMC (Mesonotal Macrosetal Count): the number of erect macrosetae on mesonotum to one side of sagittal plane.

PFL (Profemur Length): the maximum length of the profemur in lateral view.

PFW (Profemur Width): the maximum width of the profemur in lateral view.

PW (Pronotal Width): the maximum width of the pronotum in dorsal view.

PDH (Propodeum Height): height of the propodeum as measured in lateral view from the base of the metapleuron to the maximum height of the propodeum.

PMC (Pronotal Macrosetal Count): the number of erect macrosetae on pronotum to one side of sagittal plane.

SL (Scape Length): the maximum length of the antennal scape excluding the condylar bulb.

SMC (Scape Macrosetal Count): the number of erect macrosetae on the scape. Scape macrosetae can be difficult to count and the scape may need to be rotated to get an accurate count. This count does not include the terminal cluster of setae often found around the joint of the scape and the funiculus.

TL (Total Length): HL+WL+GL.

WL (Weber’s Length): in lateral view, the distance from the posteriormost border of the metapleural lobe to the anteriormost border of the pronotum, excluding the anterior cervical flange.

All measurements were taken with a Leica microscope using an orthogonal pair of micrometers, recorded to the nearest 0.001 mm, and rounded to two decimal places for presentation. Samples with missing data were dropped or species averages were inserted to the missing data (these alternative treatments made no difference to final results or conclusions; results shown for reduced dataset). We included nine nominal species in the morphometric dataset including eight samples of the RCA from Texas, Louisiana, and Mississippi, seven samples of indeterminate species designation (*N. cf. pubens*) from Florida and the Caribbean, as well as the *N. pubens* and *N. fulva* type material (Table 1, Table S1).

All morphometric analyses were carried out with the adegenet 1.3–4 package [31], [32] implemented in R 2.15.0 [33]. *K*-means clustering was conducted with all 15 principal components to sort samples into prior groups. Four clusters were chosen for subsequent discriminant analysis of principal components (DAPC) [34]. DAPC was conducted with five principal components, which explained 99.9% of the variation, and three discriminant functions were retained. To avoid over-fitting during discrimination using DAPC, we estimated the optimal number of principal components using the *optim.a.score* function in adegenet. The *a*-score measures the proportion of successful reassignments of the DAPC analysis compared to *k*-means clustering (observed discrimination) and random clustering (random discrimination). Hence, it measures the trade-off between the power of discrimination and over-fitting by using too many principal components in the analysis.

Since *k*-means clustering was able to distinguish far fewer groups than nominal species present in the morphometric dataset, we sought to further investigate how clear-cut group membership was relative to our species identifications. Hence, we repeated the DAPC, grouping samples by the ten species. For this analysis, we considered the RCA and the *N. cf. pubens* samples which we could not unequivocally assign to *N. pubens* or *N. fulva* as a distinct species. Again, we used the *optim.a.score* function to choose the optimal number of principal components (n.pca = 5). Membership assignment probabilities to each species based on retained discriminant functions were compared to the groups identified by *k*-means.

### Molecular Phylogenetic Analysis

We generated DNA sequence data from one mitochondrial gene, cytochrome oxidase I (COI), and five nuclear genes: elongation factor 1-α F1 (EF1α-F1), elongation factor 1-α F2 (EF1α-F2), wingless, carbomoylphosphate synthase (CAD) and arginine kinase (argK). Primers and PCR protocols follow LaPolla et al. [20] except that the current study also included extended fragments from CAD and COI as well as the additional gene argK. Primers for the new CAD and argK fragments are from Ward et al. [35] while those for the new COI fragment (Jerry/Pat) are from Simon et al. [36]. PCR products were sequenced using the same primers used for amplification via automated Sanger sequencing. Sequence chromatograms for each fragment were assembled and edited using Sequencher v.4.10 and combined into a phylogenetic matrix using MacClade [37]. The CAD, argK, and EF1α-F1 fragments contain introns (1,485 total bp). Excluding intronic areas, which could not be aligned confidently, or excluding all introns did not change the topology resulting from phylogenetic analyses. Only the results using the coding (exonic) sequences are presented in the following. Of the total 4,669 included nucleotide positions from all genes, 917 were variable and 594 parsimony informative. For the molecular dataset, we sampled two Zatania species as outgroups and 18 nominal Nylanderia species [20] (Table 1, Table S2). The latter includes several specimens of N. fulva and one specimen of N. pubens to which we could unambiguously assign species identities based on male morphology. Additionally, we sequenced RCA samples from Texas and Louisiana and several additional specimens of indeterminate species designation (N. cf. pubens) from the Caribbean and Florida. All sequences generated are new to this study except for some fragments previously published by LaPolla et al. [20]. Taxa and GenBank accession numbers are listed in Table S2.

Sequences were aligned using MUSCLE 3.8 [38]. All phylogenetic analyses were conducted on individual loci and the concatenated dataset. Maximum likelihood (ML) trees were bootstrapped (BS; 100 replicates) using PhyML 3.0 [39], [40] using unpartitioned datasets. Models of nucleotide substitution were estimated using 95% confidence set of models under the Bayesian Information criterion implemented in jModeltest 2.0 [41]. Bayesian MCMC tree searches were conducted using MrBayes 3.2.1 [42], [43]. Models of nucleotide substitution were estimated during the Bayesian MCMC analysis by sampling across the entire general time reversible (GTR) model space [43], thus removing the need for a priori model testing. For each analysis, two independent runs were conducted with 4 chains each (one cold and three incrementally heated, temp = 0.02–0.05). To avoid upwardly biasing tree length, we followed the recommendations of Marshall [44] and Brown et al. [45] by using a prior with a shorter mean (unconstrained exponential prior with λ estimated according to Brown et al. [45] based on ML tree lengths) as well as incorporating a whole-tree-scaling move during the MCMC. All other parameters were left at default values. We uncoupled substitution rates, state frequencies, rate variation across sites (α), and proportion of invariable sites across genes during MCMC runs, but kept topology and branch lengths linked. Because the runs converged very rapidly, chains were only run for one to three million generations for each individual locus and 10 to 20 million generations for the concatenated dataset. The chains were sampled every 1,000^th^ generation. Generally, the first 10% of the chains were discarded as burnin and the remaining 90% trees were kept to summarize model parameters, tree topology, branch lengths, and posterior probabilities (PP) of branch support. Convergence was assessed by measuring average standard deviations of split frequencies (<0.01), potential scale reduction factor (PSRF) (∼1.0), plateauing of log-likelihood values, and effective samples sizes (ESS) (>200) using MrBayes, Tracer 1.5 [46], and AWTY [47].

## Results and Discussion

**Figure 2:**
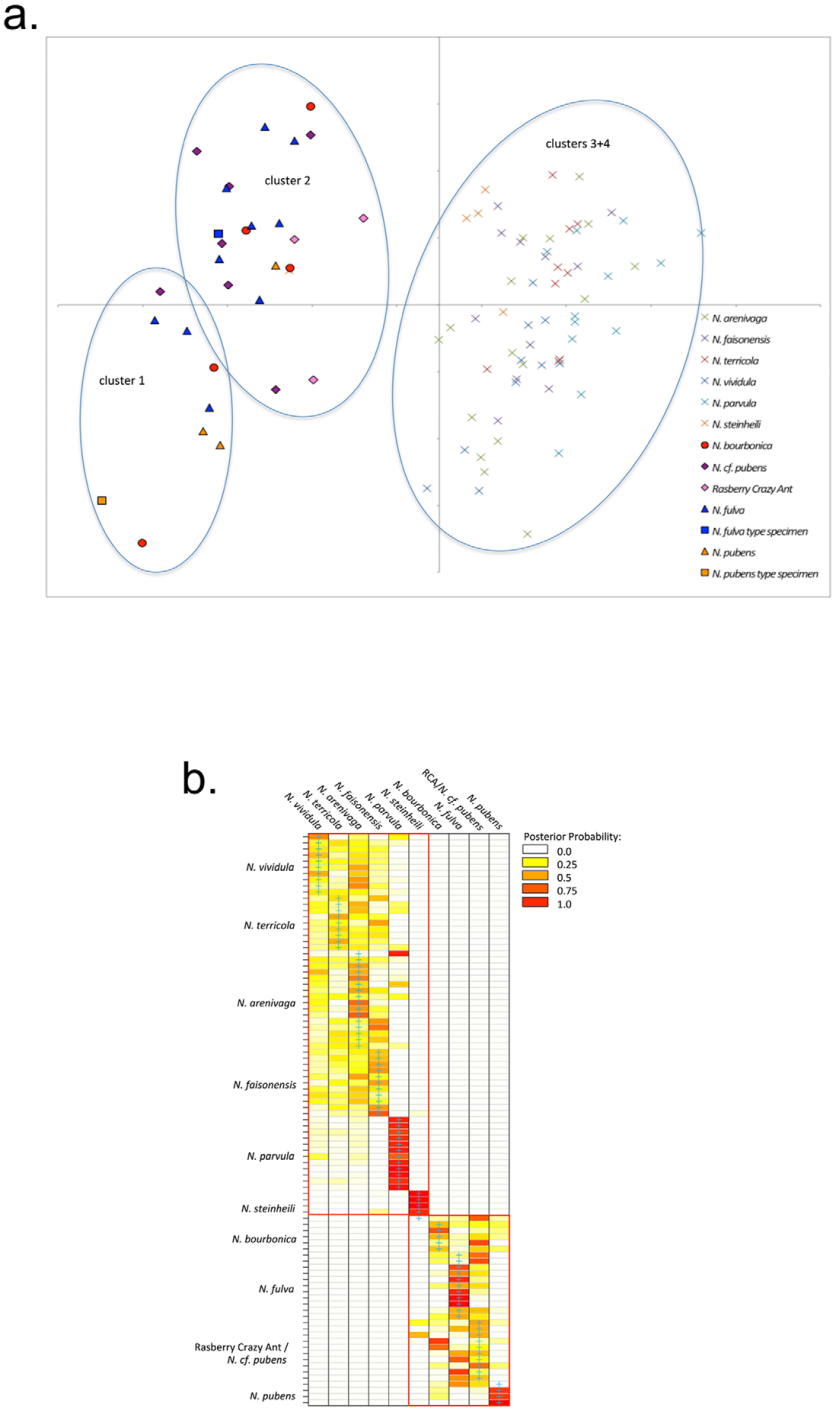
Results of the discriminant analysis of principal components (DAPC) of 14 morphometric measurements. Species are color-coded. a) Scatter plot of the first two discriminant functions based on *k*-means clustering. Variable contribution (>10% loading) to the first axis is primarily scape length, profemur length, gaster length, total length, and Weber’s length, whereas gaster length, mesonotum setae count, and total length contribute to the second axis. b) Heat map of assignment probabilities of individual samples (rows; order identical to Table S1) to species (columns) based on discriminant functions. Blue crosses indicate group membership as assigned by the authors (i.e. species membership). Cluster assignment based on *k*-means clustering is shown by red rectangles (lower right: clusters 1+2; upper left: clusters 3+4).

Here, we identify the nominal species of the RCA using morphometric and DNA sequence data. First, we applied DAPC to 14 morphological measurements of *Nylanderia pubens*, *N. fulva, N. steinheili* (which are all Neotropical), the RCA, *N. cf. pubens*, five native North American species, and the Old World invasive *N. bourbonica* (Fig. 2). *K*-means clustering identified four strongly differentiated clusters, clearly discriminating all North American *Nylanderia* species from the RCA. The first split (*k* = 2), which is associated with the largest decrease in the BIC, separates all *N. fulva*, *N. pubens*, RCA, *N. cf. pubens*, and *N. bourbonica* samples, plus a single *N. steinheili* individual (clusters 1+2) from the remaining *N. steinheili* specimens and all specimens of *N. parvula*, *N. vividula*, *N. terricola*, *N. arenivaga*, and *N. faisonensis* (clusters 3+4). The next two splits (*k* = 3 and *k* = 4) again divide each of these two clusters, but not along species lines.

Although the *k*-means clusters are well separated by DAPC, generally for our dataset *k*-means clustering and DAPC are unable to clearly differentiate nominal species of *Nylanderia* (Fig. 2a). Despite the failure of the *k*-means clustering to follow strict taxonomic lines, species mainly occupy distinct, yet partially overlapping, areas in the scatterplot of the DAPC. While most species are only marginally separated at best, clusters 1+2 and clusters 3+4 are quite distinct and separated along the first discriminant function. Importantly, the RCA (and *N. cf. pubens* are completely contained within the *N. fulva*, *N. pubens*, and *N. bourbonica* group (clusters 1+2).

To better interpret cluster membership and the power of *k*-means clustering to group samples, we repeated the DAPC using our species assignments (Fig. 2b). Based on the heat map of assignment probabilities one can distinguish three to five groups within the ten species, which agrees with the number of clusters detected by *k*-means clustering. However, only the first split (*k* = 2) is clearly visible in the heat map (Fig. 2b). Subsequent *k*-means splits (*k* = 3 and 4) are not discernable in the assignment probabilities. Individuals of three species are correctly assigned to their respective species – all *N. parvula* and all but one *N. steinheili* and *N. pubens* are assigned with high posterior probability (PP≥0.70). The remaining samples are separated into two groups (basically the *k*-means clusters), their assignment probabilities more or less equally distributed across species within a given group, especially in the *N. vividula*, *N. terricola*, *N. arenivaga*, and *N. faisonensis* cluster. Importantly, the RCA samples are generally assigned to the *N.fulva* and *N. bourbonica* cluster with very high cumulative probabilities (PP≥0.85), with the assignment probability to any species within that cluster varying on average PP≈0.18–0.33. This suggests that while *k*-means clustering is incapable of clearly delineating species within our dataset, DAPC is quite powerful in delimiting several species, despite making some mistakes. Our morphometric analysis comes to a similar conclusion from previous authors [21], [26] who were unable to clearly align the RCA with *N. pubens* using different methodologies. Our morphometric data suggest that *N. fulva* and *N. bourbonica* are candidate species for the identity of the RCA.

**Figure 3:**
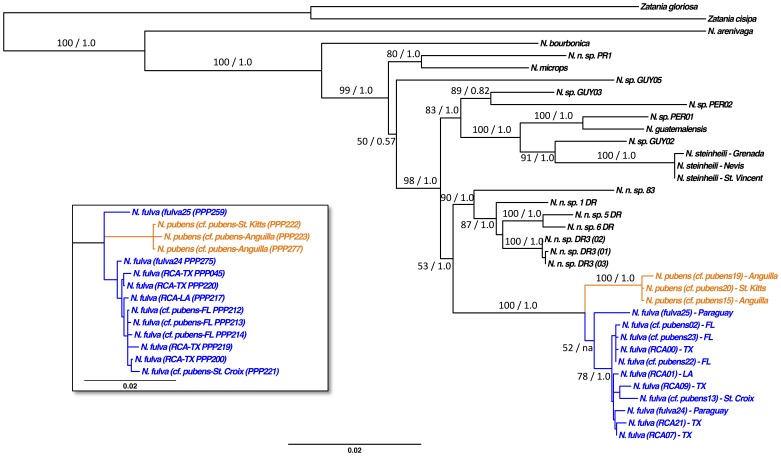
Phylogenetic tree estimation of six concatenated loci. Shown is the maximum likelihood phylogeny. Branch support values (maximum likelihood bootstrap and Bayesian posterior probabilities [BS/PP]) are shown except on the very short branches. The inset shows the unresolved Bayesian analysis for the *Nylanderia fulva* complex. *Nylanderia fulva* samples are shown in blue, *N. pubens* sample are indicated in orange.

Second, we applied molecular phylogenetic analyses to identify the RCA (Fig. 3) using one mitochondrial and five nuclear loci (∼4.7 kb total, excluding introns; GenBank accession numbers, Table S2). The molecular dataset and resulting trees are deposited in Treebase (accession number 12759). *Nylanderia fulva* and *N. pubens* together form a strongly supported clade (PP = 1.0; BS = 100) that is clearly distinct from other species, including *N. bourbonica* (which itself is an introduced species originating from the Old World tropics) and *N. steinheili*, which are rather distantly related. Importantly, all samples we initially classified as RCA or *N. cf. pubens* are also placed within this clade. The placement of *N. pubens* with two Caribbean *N. cf. pubens* samples is strongly supported (PP = 1.0; BS = 100; hereafter referred to as the *N. pubens*-clade), leading us to consider all three samples as unequivocal *N. pubens*. In the ML analysis, the *N. pubens*-clade is sister taxon to a *N. fulva*-clade, although with low statistical support (BS = 52) due to the uncertain placement of a Paraguayan *N. fulva* sample (*fulva*25). In the Bayesian analysis (Fig. 3 inset) this uncertain placement is unresolved, and the *N. pubens-clade,* the *N. fulva*-clade, and *fulva*25 sample form an unresolved polytomy. However, the remaining *N. fulva*-clade samples are recovered as monophyletic with good support (PP = 0.99; BS = 78). This clade includes all samples initially considered to be RCA, *N. cf. pubens* from Florida and the Caribbean, and unequivocal *N. fulva* from Paraguay and the Caribbean. These sequences are interspersed and separated by very short branches, suggesting conspecificity of the RCA and the Floridian *N. cf. pubens* with *N. fulva*. Separate analyses of each individual locus are all concordant with this result in placing a highly supported *N. pubens*-clade within or as sister-group to the *N. fulva*-clade, indicating problems of the dataset to resolve the split of *N. pubens* and *N. fulva* due to incomplete lineage sorting or lacking phylogenetic signal. These sequences are interspersed and separated by very short branches, suggesting conspecificity of the RCA and the Floridian *N.* cf. *pubens* with *N. fulva* [48].

In addition to the phylogenetic analyses, shared haplotypes (including intronic sequences) between samples lend support to our identification of the RCA and Floridian *N. cf. pubens* as *N. fulva*. First, not surprisingly given the results of the phylogenetic analyses, the three *N. pubens*-clade samples share some haplotypes at all loci, suggesting conspecificity. Importantly, these samples never share haplotypes with any other samples, suggesting they are reproductively and evolutionarily distinct. Second, *wingless*, *elongation factor-1a-F2*, and *elongation factor-1a-F1* haplotypes are extensively shared between all members of the *N. fulva*-clade, including with *fulva*25 at *wingless*. This clear pattern is expected under conspecificity of the RCA (and *N. cf. pubens* from Florida) with *N. fulva*.

Even though the molecular data unequivocally identify the RCA as *N. fulva*, it is perhaps a bit surprising that the phylogenetic analyses did not recover *N. fulva* as monophyletic with strong support. However, there are several, not mutually exclusive explanations for this, which do not detract from our confidence of species identification of the RCA. First, *N. fulva* and *N. pubens* appear to be young species, which have not yet fully achieved reciprocal monophyly at all loci [49], [50], [51]. Species tree methods, which account for incomplete lineage sorting, would be a more appropriate method to analyze such data [52]. However, given our goal in this study, we deemed such approaches inappropriate since they generally require samples to be correctly identified (but see O’Meara [53]). Second, nominal *N. fulva* could constitute a species flock of closely related cryptic species and our sample size was insufficient to accurately describe “intraspecific” variation. Even in this extreme case, our current identification of the RCA as nominal *N. fulva* would hold until a species delimitation study (e.g., [16], [54], [55], [56]) and identification of the source localities of the invasive populations (e.g., Suarez et al. [57]; Tsutsui et al. [58]; Caldera et al. [59]; Ascunce et al. [60]) would prove otherwise. Our current sampling is not extensive enough to test for any of these possibilities and it would be beyond the scope of the current study. However, a rigorous, integrative species delimitation project of the *N. fulva* complex (which includes *N. pubens*) is currently being conducted by the authors (JSL and RJK) within the scope of a general revision of New World *Nylanderia* species. In any case, our identification of the RCA (and North American *N. cf. pubens*) as nominal *N. fulva* holds.

**Figure 4:**
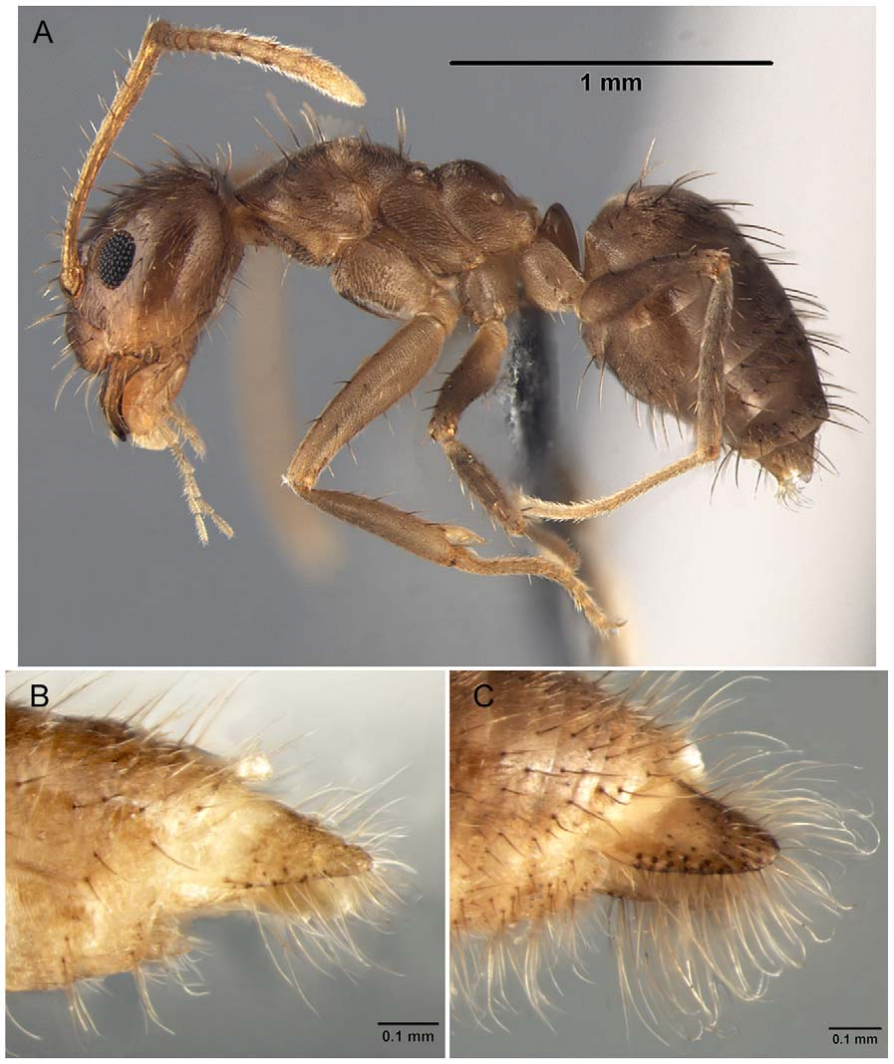
A) worker of *Nylanderia pubens* in lateral view; B) male *N. fulva* paramere in lateral view; C) male *N. pubens* paramere in lateral view.

There has been widespread misidentification of *Nylanderia fulva.* Within museum collections, misidentifications are common given the morphological similarities of the workers within the genus overall, as well as because of uncertainties regarding species boundaries. Until recently [30], [61] there has been little revisionary systematics work on *Nylanderia*. Thus in the literature and museum collections names have generally been haphazardly applied, without reference to or consideration of type specimens. There are a few species that have variously been considered as potentially confused with *N. fulva*. This partly reflects the taxonomic uncertainty among species in the genus, rather than the fact that these species are closely related, but we review those species here to help clarify the taxonomic identities pending the completion of on-going New World *Nylanderia* revisionary work by JSL and RJK. *Nylanderia fulva* can be separated from *N. guatemalensis* and *N. steinheili* based on the fact that these two species only possess dorsal mesosomal pubescence (*N. fulva* has a fully pubescent mesosoma). Among the other three species which all possess a fully pubescent mesosoma, *N. bourbonica* can be separated based on color as it is typically dark brown to nearly black, whereas *N. fulva* and *N. pubens* workers are brown to light brown. Unfortunately, workers of *N. fulva* and *N. pubens* are indistinguishable from each other, making use of male morphology crucial for distinguishing these species morphologically. Examination of the parameres provides a strong distinguishing character between the two species (Fig. 4), something already noted by Trager [27]. In *N. fulva* the parameres are more triangular in profile, less sclerotized, and possess fewer macrosetae originating from the paramere margin (Fig. 4B). Conversely, *N. pubens* parameres are rounded in profile, well sclerotized, and have many long macrosetae originating from the paramere margin in a dense, fan-like configuration in lateral view (Fig. 4C).

Having identified the invasive RCA as *N. fulva*, control, management, and study of these pests may proceed unencumbered by taxonomic uncertainty. Given the uncertainty of worker-based identifications of *N. fulva* and *N. pubens* most publications that involve either of these species are suspect; they may not involve the species listed in the publication, including the possibility that they are neither *N. fulva* nor *N. pubens* and are an entirely different *Nylanderia* species. It appears at this time that *N. pubens* is restricted to the Caribbean region. This species has been reported to be relatively to be relatively common in southern Florida in the 1950’s –1970’s [27], where it was also most recently found in 1994 (M. Deyrup, pers. comm. to JSL). It is not known whether these populations still persist today. Since we show that samples from northern Florida initially considered to be *N. cf. pubens* are actually *N. fulva* and given the invasive nature of *N. fulva*, we hypothesize that most or even all alleged occurrences of *N. pubens* in Florida (Fig. 1) are misidentified *N. fulva*. This would not be surprising, since the distribution shown in Figure 1 is solely based on worker identifications (D. Oi, pers. comm. to DG). We also suspect that *N. pubens* may not have good invasive capabilities compared to *N. fulva*, given the currently rapidly expanding distribution of *N. fulva* in the United States and lack of *N. pubens* in our samples from northern Florida. It will require much better sampling of molecular data or male samples from throughout Florida to test our hypothesis. Currently, the Caribbean is likely the only place where *N. fulva* and *N. pubens* are sympatric and therefore the only region where identifications of workers will be difficult. If we are correct concerning the distribution and inability of *N. pubens* to become a pest, then the population explosions attributed to *N. pubens* that plagued the Caribbean from 19^th^ century Bermuda [62] to the recent outbreak on St. Croix [63] and in southern Florida [28] may very well have been *N. fulva* instead of *N. pubens*. *Nylanderia fulva* is known to be an invasive ant [64], most recently from Colombia where an outbreak occurred after this species was apparently introduced to control leafcutter ants and venomous snakes [65].

Therefore outbreaks may be common in *N. fulva* and should be expected in all inhabited areas, at least in the putative invasive part of its range. The current reported distribution of *N. fulva* in the United States is still patchy (Fig. 1), but the pattern suggests that it will be able to invade the entire Gulf Coast, if it has not already done so. To accurately predict its potential range will require more detailed descriptions of its ecology, natural history, and distribution in its native range which can then be used to inform predictive environmental niche modeling [66], [67], [68], [69]. However, the native range of *N. fulva* must first be identified and, ideally, the source population(s) of the invasive populations must also be known. While this information is still lacking, it is highly likely that *N. fulva* is native to South America, probably southern South America (the type locality is in Brazil). Like other notorious invasive ants, e.g., *Solenopsis invicta* [59], [60] or *Linepithema humile* [57], [58], it is possible that *N. fulva* could be yet another ant from the greater Paraná drainage that has become invasive elsewhere [70], [71]. Detailed phylogeographic and population genetic studies based on broad and extensive sampling across the entire range of the species will help address these issues and provide the basis for effective management of *N. fulva* in North America [8], [72], for example through the introduction of co-evolved biological control agents (e.g., Caldera et al. [59]; Poinar et al. [73]; Vasquez et al. [74]).

Research can now focus on this species’ population dynamics, ecology, natural history, and identification of its native range to better understand the causes and consequences of such rapid population growth. This endeavor would not have been possible without the collection-based resources and taxonomic expertise present in natural history museums, underscoring their value for both basic and applied research [75], [76].

## Supporting Information

Table S1 **Morphometric data.** For specimens where GPS coordinates were not provided on the label (indicated by an asterisk), when possible we estimated the GPS data based on the collection information. Specimens highlighted in red were excluded from DAPC due to missing data points.
(XLS)

Table S2 **Molecular data and GenBank accession numbers.**
(XLSX)
